# 597 A Review of the Centralization of Burn Care: A “Hub and Spoke” Model

**DOI:** 10.1093/jbcr/iraf019.226

**Published:** 2025-04-01

**Authors:** Anna Dargan, Paul Baker, Richard Wong She

**Affiliations:** Middlemore National Burns Centre, Middlemore Hospital; National Burn Centre of New Zealand, Middlemore Hospital; National Burn Centre of New Zealand, Middlemore Hospital

## Abstract

**Introduction:**

The centralization of burn care involves consolidating specialized burn treatment services into a few highly equipped centers, aimed at improving patient outcomes by concentrating expertise and resources in specific locations. The National Burn Center (NBC) was created in 2006 to serve a population of 4.185 million people. Our current total catchment population is 5.1 million people spread over 268, 021 km2, compared to the average population of a US state of 5.7 million over 182, 949 km2. The NBC is located in the largest metropolitan area and in addition to loco-regional smaller burn injuries also accepts referrals of “severe” burn injuries (e.g. >30% total burn surface area (TBSA) from across the country.

Challenges include geographic access, particularly for patients from rural or underserved areas, ensuring equitable access, and resource management when balancing the National Center needs with the loco-regional burns service provision. The “hub and spoke” model was implemented to overcome some of these challenges. This model involves centralizing complex and resource-intensive healthcare services at a “ hub” facility (NBC), and maintaining a network of “ spoke” facilities, (regional burns units - RBUs), and relies on strong collaboration between the hub and spoke facilities including clear referral pathways and support/training for regional centers.

**Methods:**

We reviewed the 7255 admissions to the NBC over a 17-year period, and identified changes in the trends of referrals, treatment approaches and outcomes. All patients received a multi-disciplinary team approach throughout their stay.

**Results:**

A significant changes in the burn treatment model was the regular use of a biodegradable temporizing matrix (BTM) introduced in 2018, which has contributed to the noted increase in survivability of burns >70% TBSA but also the increased length of hospital stay in 30-50% TBSA group. There was a decrease in the percentage of annual out-of-region transfers to the NBC, in keeping with a tighter referral criterion, and improved support and expertise in the RBUs. We also noted a persistent over-representation of Maori and Pasifika patients in our cohort compared to the population.

**Conclusions:**

In conclusion, this model provides significant benefits, including improved outcomes, specialized multidisciplinary care, and a focus on research and innovation. However, addressing challenges related to equitable access remains crucial. By optimizing these systems, centralized burn care can significantly enhance both the immediate and long-term recovery outcomes for individuals with burn injuries.

**Applicability of Research to Practice:**

This paper explores the evolving practices in burn management that have emerged since the establishment of a centralized National Burn Center, as well as the ongoing development of new models of care for burn treatment.

**Funding for the Study:**

N/A

